# The impact of cryopreserved sperm on intrauterine insemination outcomes: is frozen as good as fresh?

**DOI:** 10.3389/frph.2023.1181751

**Published:** 2023-05-31

**Authors:** Panagiotis Cherouveim, Stylianos Vagios, Karissa Hammer, Victoria Fitz, Victoria S. Jiang, Irene Dimitriadis, Caitlin R. Sacha, Kaitlyn E. James, Charles L. Bormann, Irene Souter

**Affiliations:** ^1^ Massachusetts General Hospital Fertility Center, Department of Obstetrics, Gynecology, and Reproductive Biology, Division of Reproductive Endocrinology and Infertility, Massachusetts General Hospital and Harvard Medical School, Boston, MA, United States; ^2^ Department of Obstetrics and Gynecology, Tufts Medical Center, Boston, MA, United States; ^3^ Division of Reproductive Endocrinology and Infertility, University of Massachusetts Chan Medical School, Worcester, MA, United States; ^4^ Deborah Kelly Center for Outcomes Research, Department of Obstetrics, Gynecology, and Reproductive Biology, Massachusetts General Hospital and Harvard Medical School, Boston, MA, United States

**Keywords:** cryopreservation, fresh, frozen, sperm, intrauterine insemination, outcomes

## Abstract

**Introduction:**

Frozen sperm utilization might negatively impact cycle outcomes in animals, implicating cryopreservation-induced sperm damage. However, *in vitro* fertilization and intrauterine insemination (IUI) in human studies are inconclusive.

**Methods:**

This study is a retrospective review of 5,335 IUI [± ovarian stimulation (OS)] cycles from a large academic fertility center. Cycles were stratified based on the utilization of frozen (*FROZEN*, *n* = 1,871) instead of fresh ejaculated sperm (*FRESH*, *n* = 3,464). Main outcomes included human chorionic gonadotropin (HCG) positivity, clinical pregnancy (CP), and spontaneous abortion (SAB) rates. Secondary outcome was live birth (LB) rate. Odds ratios (OR) for all outcomes were calculated utilizing logistic regression and adjusted (adjOR) for maternal age, day-3 FSH, and OS regimen. Stratified analysis was performed based on OS subtype [*gonadotropins*; *oral medications* (*OM*): clomiphene citrate and letrozole; and *unstimulated/natural*]. Time to pregnancy and cumulative pregnancy rates were also calculated. Further subanalyses were performed limited to either the first cycle only or to the partner's sperm only, after excluding female factor infertility, and after stratification by female age (<30, 30–35, and >35 years old).

**Results:**

Overall, HCG positivity and CP were lower in the *FROZEN* compared to the *FRESH* group (12.2% vs. 15.6%, *p* < 0.001; 9.4% vs. 13.0%, *p* < 0.001, respectively), which persisted only among *OM* cycles after stratification (9.9% vs. 14.2% HCG positivity, *p* = 0.030; 8.1% vs. 11.8% CP, *p* = 0.041). Among all cycles, adjOR (95% CI) for HCG positivity and CP were 0.75 (0.56–1.02) and 0.77 (0.57–1.03), respectively, *ref: FRESH.* In *OM* cycles, adjOR (95% CI) for HCG positivity [0.55 (0.30–0.99)] and CP [0.49 (0.25–0.95), *ref.*: *FRESH*] favored the *FRESH* group but showed no differences among *gonadotropin* and *natural* cycles. SAB odds did not differ between groups among *OM* and *natural* cycles but were lower in the *FROZEN* group among *gonadotropin* cycles [adjOR (95% CI): 0.13 (0.02–0.98), *ref.*: *FRESH*]. There were no differences in CP and SAB in the performed subanalyses (limited to first cycles or partner's sperm only, after excluding female factors, or after stratification according to female age). Nevertheless, time to conception was slightly longer in the *FROZEN* compared to the *FRESH* group (3.84 vs. 2.58 cycles, *p* < 0.001). No significant differences were present in LB and cumulative pregnancy results, other than in the subgroup of *natural* cycles, where higher LB odds [adjOR (95% CI): 1.08 (1.05–1.12)] and higher cumulative pregnancy rate (34% vs. 15%, *p* = 0.002) were noted in the *FROZEN* compared to the *FRESH* group.

**Conclusion:**

Overall, clinical outcomes did not differ significantly between frozen and fresh sperm IUI cycles, although specific subgroups might benefit from fresh sperm utilization.

## Introduction

In the United States, approximately 12%–18% of women attempting conception struggle with infertility (1). A variety of fertility treatments are available, and intrauterine insemination (IUI), with or without ovarian stimulation (OS), is often considered a first-line treatment for many patients. Despite its wide utilization in the United States, there is no national reporting system for IUI cycle outcomes. Some data are available from the European Society of Human Reproduction and Embryology (ESHRE), which reports outcomes on over 200,000 IUI cycles annually (2).

Multiple patient characteristics and cycle-related factors, including semen parameters, maternal age, and type of OS protocol, have been evaluated for their impact on IUI cycle outcomes (3). However, the impact, if any, of sperm cryopreservation on the outcome of IUI cycles remains unclear. In 1992, Subak et al. (4) found that fecundity rates in IUI cycles (with or without OS) utilizing frozen sperm did not differ significantly from that of cycles utilizing fresh ejaculated sperm. However, the study, published over 30 years ago, was mostly limited by the small sample size (less than 200 cycles included) and the lack of information on administered OS regimens. Since then, ultrasound monitoring, OS protocols, and sperm processing and cryopreservation technologies have evolved, necessitating further studies to evaluate the impact of these techniques on clinical outcomes. Some relevant reports exist evaluating not the possible impact of sperm cryopreservation on the outcome of an IUI cycle, but rather the impact of sperm origin on it, mostly by comparing donor's to partner's ejaculated sperm, with the former being almost always cryopreserved and the latter usually fresh (2, 5, 6).

Although animal studies suggest less favorable cycle outcomes with frozen compared to fresh sperm utilization, human Assisted Reproductive Technology (ART) data remain inconclusive. Two meta-analyses reported no differences on cycle outcomes between frozen and fresh sperm in the *in vitro* fertilization (IVF) setting (7, 8). Nevertheless, these studies included men with severe male factor infertility utilizing intracytoplasmic sperm injection (ICSI) and surgically extracted sperm, both in the frozen and fresh sperm groups. On the contrary, in a study among men with neurological or psychological anejaculation, outcomes evaluated (embryo quality and pregnancy rates) were less favorable with frozen compared to fresh sperm acquired with electroejaculation (9). Similarly, data from animal studies suggest that outcomes were worse with frozen compared to fresh sperm among a wide variety of animals including sheep, dogs, and horses (10–12).

Given the scarcity of data and the lack of consensus on this topic, we aimed through the present study to evaluate whether the utilization of frozen, instead of fresh ejaculated sperm, has any impact on the clinical outcomes of IUI cycles (with or without OS).

## Materials and methods

### Study design

Data from 15,952 cycles, performed at the Massachusetts General Hospital (MGH) Fertility Center between January 2004 and December 2021, were retrospectively reviewed. Cycles with incomplete data on either cycle outcomes or type of utilized sperm were excluded, as were those cancelled prior to human chorionic gonadotropin (HCG) trigger or utilizing timed intercourse instead of an IUI. Most common causes for cycle cancellation prior to HCG trigger had to do with the woman's response to stimulation (either high or low response) and had nothing to do with insufficient sperm sample quality. Cycles included in the final analysis were stratified in two groups (*FROZEN* vs. *FRESH* ejaculated sperm). All couples coming in our practice underwent a standard fertility evaluation, as was previously described (13). In cycles utilizing frozen sperm (autologous or donor), all diagnoses were included in the *FROZEN* group. Since most common indication for IUI in the *FROZEN* group was not related to the diagnosis of infertility, i.e., single mother by choice or same-sex couples, we only included cycles performed among couples with idiopathic infertility in the *FRESH* ejaculated sperm group to allow comparisons. After application of inclusion and exclusion criteria, the *FROZEN* group included 1,871 IUI cycles from 487 women, while the *FRESH* group included 3,464 IUI cycles from 1,342 women.

The study was approved by the Partners’ Healthcare Institutional Review Board.

### Andrological information

In the *FRESH* group, all couples included had diagnosis of idiopathic infertility, and, therefore, male partners had normal semen parameters by the WHO 5th edition criteria (14) and at least a postprocessing total motile count (TMC) over 1 million (the latter required by insurances to permit an insemination). The density gradient sperm preparation was utilized until September 2017 and the simple wash method afterward. The former technique included multiple centrifugation and suspension steps with the aim of yielding a highly motile purified sample of sperm, while the latter included one centrifugation step utilizing a multipurpose handling medium. Most of the sperm samples in the *FROZEN* group were from anonymous donors (96.3%) and were transferred in our practice from sperm banks of the patient's choice. In a small number of cases, partner's sperm cryopreservation was required for logistic reasons (including difficulties with ejaculation and unavailability to provide a sample on the day of the IUI procedure), to avoid cycle cancellation after HCG administration. Sperm cryopreservation in our practice was done consistently throughout the study period utilizing the TEST-yolk buffer with glycerol media method as previously described (15), with no dramatic decrease in sperm motility observed. Finally, the reported TMC represents the postprocessing TMC that was inseminated.

### Cycle protocols

In cycles with OS, stimulation was initiated on cycle day 3. Serial ultrasounds were performed to monitor response to treatment in all cycles, and when at least one follicle was ≥16 mm, recombinant HCG was used to trigger ovulation (Ovidrel 250 mg; Serono Laboratories, Inc., Norwell, MA, United States). Thirty-six hours after administering the HCG trigger, IUI was performed, utilizing either fresh or frozen sperm. Serum β-human chorionic gonadotropin levels were obtained 16 days after the IUI, and levels >6 mIU/ml were considered positive.

Electronic medical records were retrospectively reviewed, and detailed information was collected on patient, cycle, and treatment characteristics.

### Clinical outcomes

Main outcome measures included HCG positivity, clinical pregnancy (CP), and spontaneous abortion (SAB) rates. IUI outcomes were compared between women who utilized either frozen or fresh ejaculated sperm. Even though we did not have complete data on live births (LBs), secondarily, we investigated potential differences in LB rates.

Clinical pregnancy was defined as the presence of an intrauterine gestational sac at approximately 6 weeks of gestation, detected by transvaginal ultrasonography.

Spontaneous abortion was defined as the loss of a clinical pregnancy after its sonographic confirmation and before viability (defined as a pregnancy over 24 weeks).

### Statistical analysis

Categorical variables were analyzed using chi-square or Fisher's exact test, as appropriate. Numerical variables were analyzed using the Mann–Whitney U-test or the *t*-test depending on the normality of each variable's distribution.

Initially, outcomes of interest were compared between *FROZEN* and *FRESH* sperm groups while including all cycles and without taking into consideration information on OS regimens (if any used). Subsequently, in a subgroup analysis, the type of OS was considered, and the following three subgroups were identified: *gonadotropins*, *oral medications (OM)* [including clomiphene citrate (CC) and letrozole (LTZ)], and *unmedicated/natural* cycles. Outcomes of interest were then compared between *FROZEN* and *FRESH* cycles between these subgroups.

Odds ratios (OR) and their respective 95% confidence intervals (CI) were calculated using generalized estimating equations logistic regression analysis for all outcomes of interest, namely, positive HCG, clinical pregnancy, and spontaneous abortion, to allow a comparison between *FROZEN* and *FRESH* sperm groups, while accounting for multiple cycles per patient. Furthermore, ORs were adjusted (adjOR) for maternal age, day-3 FSH, and OS protocol in the main analysis, while in the subgroup analysis, adjustment was done for maternal age and day-3 FSH.

Time to pregnancy in cycles was also estimated with Kaplan–Meier curves, before and after subgroup analysis, utilizing the number of cycles as the unit of measurement. Additionally, cumulative pregnancy rates were calculated before and after OS stratification.

Further subanalyses were performed. The first was limited to first cycles only, both before and after stratification by OS type. Additional subanalyses were conducted, limited to either partner's sperm only, or after excluding female factor infertility, or stratifying by female age (<30, 30–35, and >35 years old).

Statistical significance was considered as a *p*-value <0.05. Statistical analysis was performed using STATA.

## Results

### Study population

Women's baseline characteristics are shown in Table 1*.* The most common diagnoses among women in the *FROZEN* group were in the following order: single mother and/or same-sex relationship (42.0%), followed by male gender (24.8%), and combined factors of infertility (22.6%). Most of the cycles (60.3%) in the *FROZEN* group were natural, while approximately 63% of the *FRESH* group cycles utilized gonadotropins for OS. Women in the *FROZEN* group were older, with higher BMIs, and evidence of slightly lower ovarian reserve (as manifested by day-3 FSH). Most of the cycles in the *FROZEN* group utilized donor's sperm (96.3%). As expected, mean total motile counts differed between the groups, both among all cycles and within subgroups, while there were no differences in concentration or motility (Table 1).

**Table 1:** Baseline and cycle characteristics.

(A) Baseline women characteristics (*n* = 1,829)			
Variable	Frozen (*n* = 487)	Fresh (*n* = 1,342)	*p*-value
Age (years)			
Mean (SD)	36.7 (5.1)	34.5 (3.7)	
Median (IQR)	37.0 (34.0, 40.0)	34.0 (32.0, 37.0)	<0.001
D3 FSH (U/L)			
Mean (SD)	7.8 (5.1)	7.0 (1.9)	
Median (IQR)	6.8 (5.8, 8.5)	6.8 (5.8, 8.0)	<0.001
AMH (ng/ml)			
Mean (SD)	3.2 (3.4)	3.2 (2.3)	
Median (IQR)	2.2 (1.0, 4.1)	2.6 (1.6, 4.1)	0.002
BMI (kg/m^2^)			
Mean (SD)	26.3 (5.5)	24.3 (4.4)	
Median (IQR)	25.0 (22.3, 29.2)	23.4 (21.1, 26.2)	<0.001
(B) Cycle characteristics (*n* = 5,335)			
Variable	Frozen (*n* = 1,871)	Fresh (*n* = 3,464)	*p*-value
Stimulation Used, *N* (%)			
Gonadotropins	348 (18.6)	2,177 (62.8)	
Clomiphene and letrozole	394 (21.1)	1,157 (33.4)	
Natural cycle	1,129 (60.3)	130 (3.8)	<0.001
Total motile count (million/sample)			
Mean (SD)	14.3 (10.5)	65.0 (74.3)	
Median (IQR)	11.4 (8.2, 17.5)	42.0 (19.1, 82.5)	<0.001
Concentration			
Mean (SD)	233.7 (180.7)	232.9 (183.0)	
Median (IQR)	95.5 (55.7, 434.7)	95.5 (54.1, 434.7)	0.211
Motility			
Mean (SD)	52.7 (15.1)	52.6 (15.3)	
Median (IQR)	66.0 (38.0, 66.0)	66.0 (38.0, 66.0)	0.960

FSH, follicle-stimulating hormone; AMH, anti-Mullerian hormone; BMI, body mass index.

### Outcomes

Overall, and prior to adjusting for potential confounders, HCG positivity and CP rates were lower in the *FROZEN* compared to the *FRESH* group (12.2% vs. 15.6%, *p* < 0.001; 9.4% vs. 13.0%, *p* < 0.001; for HCG positivity and CP, and for *FROZEN* vs. *FRESH,* respectively).

However, after further stratification by OS type (namely, *gonadotropins*, *OM*, and *natural/unstimulated* cycles), the above observed differences persisted only among cycles utilizing *OM* for OS. In the latter group, HCG positivity and CP were lower in the *FROZEN* compared to the *FRESH* group (HCG positivity: 9.9% vs. 14.2%, *p* = 0.030; CP: 8.1% vs. 11.8%, *p* = 0.041, for *FROZEN* vs. *FRESH,* respectively).

**Figure 1:**
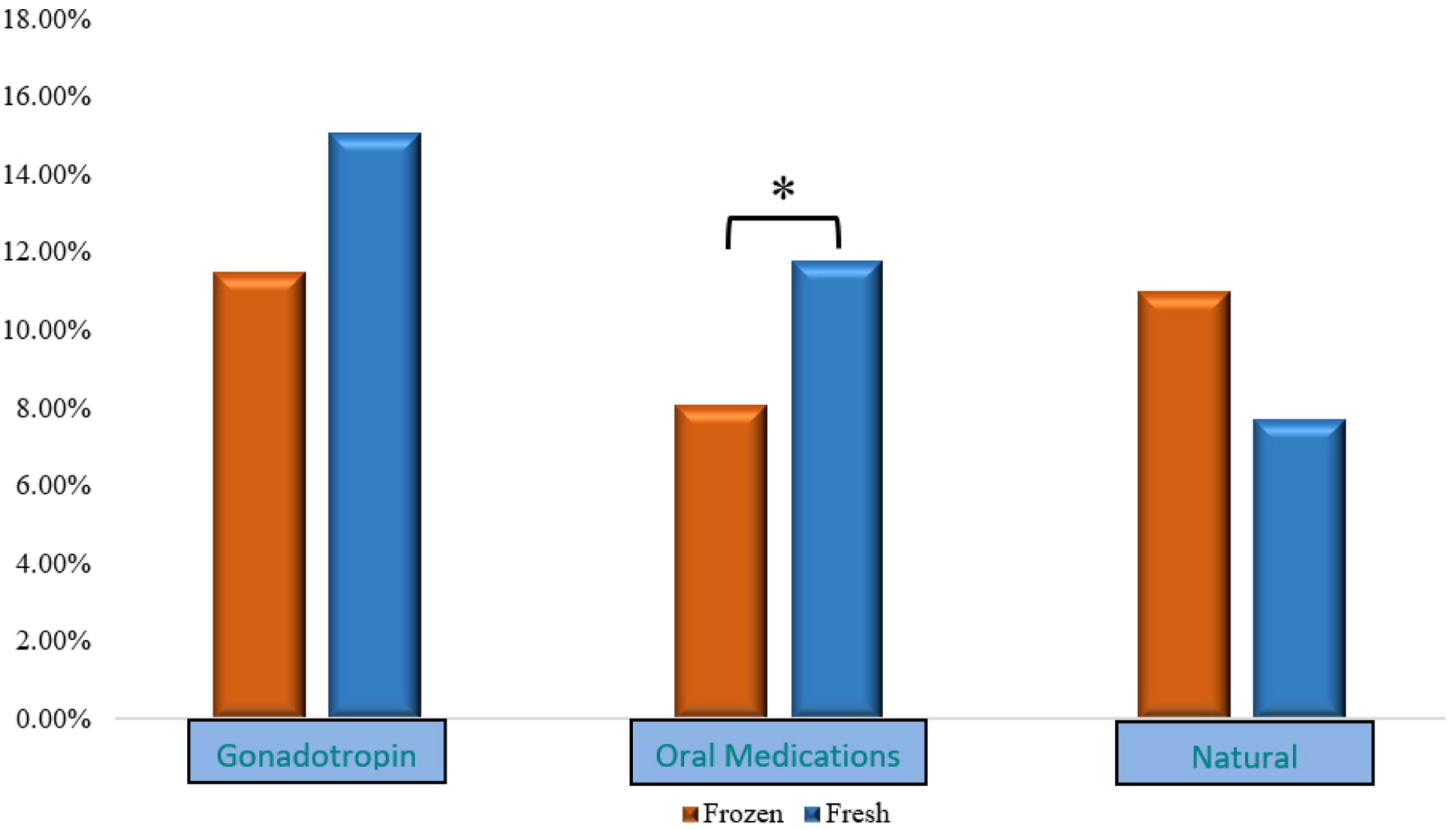
CP rates after stratification according to OS. CP, clinical pregnancy; OS, ovarian stimulation.

Figure 1 shows clinical pregnancy rates after further stratification according to OS protocol.

Regarding SAB, before adjustment, no statistically significant differences were noted between the *FROZEN* and *FRESH* groups either before or after stratification by OS type.

After adjusting for potential confounders and prior to stratifying cycles according to treatment protocols, adjORs suggested that the odds of positive HCG and CP were favoring the *FRESH* group, albeit not statistically significant [adjOR (95% CI): 0.75 (0.56–1.02), *p* = 0.07; 0.77 (0.57–1.03), *p* = 0.08; for positive HCG and CP, respectively, *FRESH* group as *ref.*]. After stratification by treatment protocol, the adjusted odds for positive HCG and CP were significantly lower in the *FROZEN* compared to the *FRESH* group only among cycles utilizing *OM*. No differences were noted in SAB rates among cycles utilizing *OM*, or in the *natural/unstimulated* subgroup but there were not enough observations in the latter to reach meaningful conclusions. Interestingly, the odds of an SAB were lower among *gonadotropin* cycles in the *FROZEN* compared to the *FRESH* group [adjOR (95% CI): 0.13 (0.02–0.98), *p* = 0.048] (Table 2), though this finding is limited by low power (only four SABs in the *FROZEN* gonadotropin group). LB and cumulative pregnancy results were similar except for higher odds for LB and higher cumulative pregnancy rate in the *FROZEN* group compared to the *FRESH* group among *natural* cycles [LB adjOR (95% CI): 1.08 (1.05–1.12); cumulative pregnancy rate: 34% vs. 15%, *p* = 0.002], which should be cautiously interpreted since there were only 10 clinical pregnancies and 2 livebirths from 57 patients in the *FRESH natural* group.

**Table 2:** Unadjusted and adjusted ORs (95% CI) for positive HCG, clinical pregnancy, and spontaneous abortion before and after subgroup analysis (*FRESH* as *ref.*).

(A) Unadjusted and adjusted^a^ ORs (95% CI) before stratification by treatment type			
	Positive HCG	CP	SAB
Unadjusted			
All cycles	0.77 (0.65–0.93)^b^	0.75 (0.63–0.91)^b^	0.92 (0.59–1.42)
First cycles	0.71 (0.52–0.96)^b^	0.75 (0.54–1.04)	1.11 (0.50–2.53)
Adjusted^a^			
All cycles	0.75 (0.56–1.02)	0.77 (0.57–1.03)	0.69 (0.32–1.50)
First cycles	0.75 (0.45–1.29)	0.79 (0.45–1.39)	0.26 (0.01–4.92)
(B) Adjusted^c^ ORs (95% CI) after stratification by treatment type			
Subgroup	Positive HCG	CP	SAB
Gonadotropin			
All cycles	0.70 (0.44–1.10)	0.65 (0.40–1.06)	0.13 (0.02–0.98)^b^
First cycles	0.44 (0.17–1.16)	0.41 (0.14–1.18)	(−)
Oral medications			
All cycles	0.55 (0.30–0.99)^b^	0.49 (0.25–0.95)*	2.24 (0.50–10.20)
First cycles	0.85 (0.30–2.39)	0.61 (0.17–2.18)	(−)
Natural/unstimulated			
All cycles	1.41 (0.64–3.07)	1.83 (0.77–4.32)	(−)
First cycles	1.86 (0.53–6.50)	5.64 (0.74–43.1)	(−)

HCG, human chorionic gonadotropin; CP, clinical pregnancy; SAB, spontaneous abortion.

^a^ Adjusted for maternal age, day-3 follicle-stimulating hormone (FSH), and ovarian stimulation (OS) protocol.

^b^ Statistically significant.

^c^ Adjusted for maternal age, and day 3 FSH.

(−): not enough outcomes.

When analysis was limited to first cycles only within each OS protocol, the unadjusted differences in HCG positivity and CP were in the same direction in cycles utilizing *OM* but did not reach statistical significance (7.6% vs. 15.5%, *p* = 0.062; 5.1% vs. 12.5%, *p* = 0.054; for the *FROZEN* and *FRESH* groups, respectively). After adjusting for potential confounders, the ORs did not suggest significant differences between the groups, neither among first cycles, nor after limiting our analysis only to the partner's sperm, nor after excluding couples with female factor infertility. As with the results of our previous analysis, where we controlled for age, instead of stratifying (Table 2), we found no statistically significant differences between the *FROZEN* and *FRESH* groups, in terms of clinical pregnancy or SAB within the age strata (<30, 30–35, and >35). A slightly higher odds for positive HCG in the *FROZEN* compared to the *FRESH* group within the age <30 stratum was noted [adjOR (95% CI): 3.67 (1.05–12.90)], but numbers are too small to allow meaningful conclusions.

**Figure 2:**
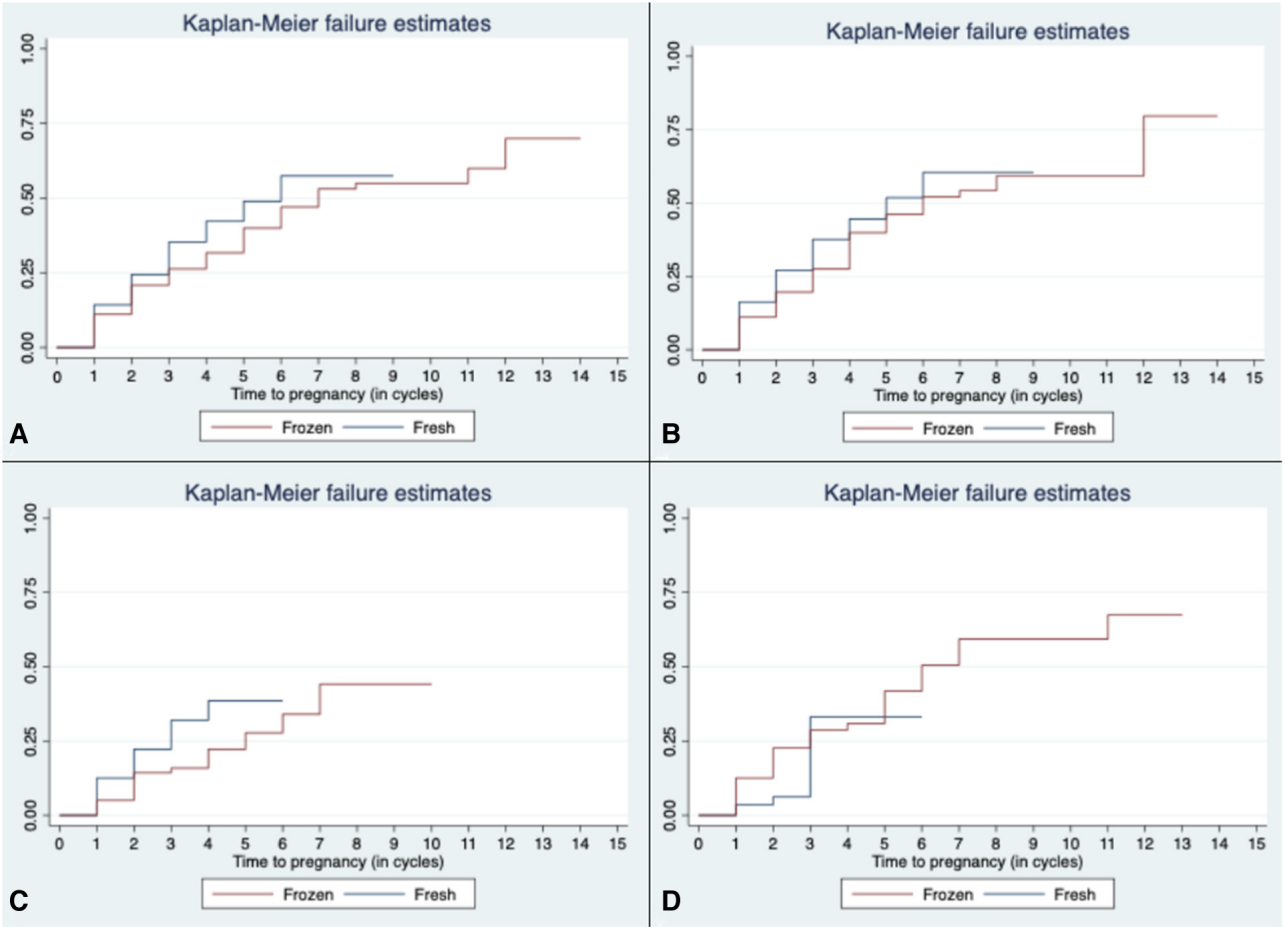
Time to pregnancy in all cycles (**A**) and according to OS protocol [(**B**) *gonadotropin*, (**C**) *OM*, and (**D**) *natural/unstimulated*]. OS, ovarian stimulation; OM, *oral medications*.

Time to pregnancy is depicted in the Kaplan–Meier curves in Figure 2. On average before subgroup analysis, women in the *FROZEN* group when compared to those in the *FRESH* group required more time to conceive (3.84 compared to 2.58 cycles, *p* < 0.001) (Figure 2A). After further stratification into OS subgroups, this difference becomes more noticeable in the *OM* subgroup (Figure 2C).

## Discussion

### Principal findings

In this study, we evaluated the impact of sperm cryopreservation on IUI outcomes. Overall, and after adjusting for potential confounders, no major difference in CP or SAB rates was noted between the *FROZEN* and *FRESH* groups. The results were unchanged when analysis was limited to first cycles only. Although women in the *FROZEN* group were older with slightly lower ovarian reserve, they might have been of more favorable prognosis with regard to a lower incidence of infertility diagnoses among them. When further stratifying cycles by OS regimen, and after adjusting for potential confounders, cycles utilizing *OM* appeared to have lower clinical pregnancy rates when frozen instead of fresh ejaculated sperm was used. Similarly, time to pregnancy seemed to be longer among the *OM* cycles that utilized frozen sperm. These findings, taken together, might suggest that among patients undergoing IUI cycles stimulated with *OM*, insemination with fresh ejaculated sperm would be preferable, if feasible. This finding needs to be further investigated and maybe of clinical importance since it may identify a subgroup of patients who would benefit from either an OS regimen modification or insemination with fresh ejaculated sperm. Nevertheless, overall, no detrimental effect from frozen sperm utilization on the outcomes of the IUI cycles was noted, similar to findings from studies performed in IVF populations.

### Results in the context of what is known

In contrast to the limited data available for IUI outcomes, there has been more substantial research in the IVF setting. In accordance with our results, two meta-analyses of ART studies in humans support no inferior results with frozen sperm (7, 8). However, in these studies, both groups included patients with severe male factor infertility and ICSI was utilized potentially masking a difference between the two groups. In a study including men with anejaculation of neurologic or psychogenic origin, lower clinical pregnancy rates were observed following ICSI with frozen compared to fresh ejaculated sperm (21.2% vs. 41.3%, *p* < 0.05). The study was limited by the small number of cycles (*n* = 96) included in their analysis (9). In the only study (4) comparing outcomes following IUIs with either frozen or fresh sperm, no significant differences were noted in conception rates. This study was limited by the small number of included cycles and the lack of information on utilized OS regimen, when applicable, and was published almost 30 years ago.

Our study was performed on a much larger number of IUI cycles and found no major difference between outcomes of cycles utilizing frozen over fresh sperm, except among the subgroup of women utilizing *OM*. Cycles utilizing frozen sperm, when compared to the ones utilizing fresh sperm, had slightly lower odds for achieving pregnancy and did so in a longer time period, an observation that persisted even after adjusting for potential confounders. Interestingly, a lower chance of SAB was noted among gonadotropin cycles utilizing frozen sperm. Although we adjusted for potential confounders, the small number of SABs in the *FROZEN* subgroup limits our ability to draw meaningful conclusions. Unfortunately, the study by Subak et al., did not permit any meaningful conclusions regarding the impact, if any, of sperm cryopreservation on the incidence of SAB mainly because SAB numbers were very small and information on OS regimens was not provided (4).

### Research and clinical implications of sperm cryopreservation

Insight into the impact of cryopreservation and thawing processes on spermatozoa and their function is provided by many *in vitro* studies (16–18). Mechanisms potentially explaining the observed differences involve disruption of sperm's functional integrity (19) and a possible adverse impact of sperm cryopreservation process on its motility (20).

A variety of factors mainly involved in the cryopreservation and thawing processes [such as cryoprotective agents (CPAs), low temperatures, and reactive oxygen species (ROS)] might be mediating the above-mentioned effects on spermatozoa, thus inflicting structural damage to the membrane or cytoskeleton, and impairing their mobility and ability to fuse with the oocyte (16–18). *In vitro*, sperm cryopreservation has been associated with damage to the cytoskeleton, DNA, and acrosome leading to adverse effects on spermatozoa's motility and viability (17). Factors potentially responsible for these changes include CPAs, which are implicated in plasma membrane and acrosome damages because of their osmotic properties (16). Furthermore, low temperatures might be inducing a hypothermic injury and thus have an irreversible adverse impact on the cell membrane (16). Moreover, ROS produced both during the cryopreservation and thawing processes might negatively affect sperm on various of its functions including decreased motility (18) and impaired ability to fuse with the oocyte (16). These *in vitro* observed effects of freezing on sperm motility and functional integrity are confirmed in animal studies, along with an impact on fertilization rates (10).

Future research, ideally large prospective cohorts, should focus on investigating the potential effects of freezing processes and determining if there are any differences in IUI outcomes between frozen and fresh sperm.

### Strengths and limitations

The major strength of our study was the large number of cycles included (more than 5,000 cycles), all from a single institution, which minimizes variability in clinical protocols and laboratory techniques. Furthermore, patient and cycle characteristics were considered when analyzing and interpreting findings, while further subanalyses were performed either limited to partner sperm or first cycles only, and after stratifying by female age, or after excluding female factor infertility yielding similar results. Finally, the results of the time-to-pregnancy analysis were in agreement with the regression analysis findings, slightly favoring fresh sperm, especially in the *OM* subgroup. However, the difference of 1.26 cycles might not be of clinical significance and might simply be explained by the fact that the TMC in the frozen sperm samples were significantly lower than those observed in the fresh ejaculated group.

Our study is not without its limitations. The study period spans over a period of 17 years and even though our practice's cryopreservation procedures have not changed, we do not have information on the Cryobanks’ cryopreservation protocols utilized for the anonymous donor samples. The overwhelming majority of the *FROZEN* group (96.3%) received IUI treatments with anonymous donor sperm, for whom we did not have access to detailed baseline information, restricting our ability for relevant analyses. Moreover, sperm from anonymous fertile donors might be of better quality and have higher tolerability to cryopreservation compared to infertile men. Despite including a large number of cycles, groups differed some in diagnosis. A good percentage of women in the *FROZEN* group were undergoing IUI for non-infertility-related reasons (single mother, same-sex relationship), while women in the *FRESH* group suffered from idiopathic infertility. As expected, TMC differed between the two groups again favoring the *FRESH* group. Nevertheless, the above-mentioned characteristics are intrinsic to both patient populations across almost all fertility practices. In clinical reality, most women opting to utilize frozen sperm utilize donor and not partner sperm, while there are very few reasons for which a partnered woman would utilize frozen partner's sperm, a fact that makes the design of the ideal study extremely challenging. Nevertheless, women opting to utilize frozen donor sperm do want to know whether their chances of conception are inferior to those of partnered women utilizing fresh ejaculated sperm. It is reassuring that despite women being slightly older in the *FROZEN* group, with evidence of lower ovarian reserve, less frequently utilizing OS, and often inseminated with lower TMC sperm, no clinically significant difference in the success was noted between the groups, even after adjusting for potential confounders. Results, including LBR, were also similar when limiting our analysis to cycles utilizing partner's sperm only or after excluding female factor infertility cases, controlling or stratifying by female partner age. Results on LBR should be interpreted with caution, since consistent information on LBR was unavailable on 19% of women that delivered in outside hospitals. With respect to the generalizability of the findings, the impact of a state insurance mandate for infertility coverage and the academic setting of our practice might need to be taken into consideration, although regarding the latter the population is diverse and of variable socioeconomic background.

## Conclusion

Our study compared IUI outcomes between frozen and fresh sperm. Similar to previously published studies, our results do not suggest a significant difference in IUI outcomes between frozen and fresh sperm. However, there is some evidence suggesting potential differences, favoring fresh sperm, in cycles stimulated with either clomiphene or letrozole. This information should be used cautiously during patient counseling and should take into account the various confounding factors. Future research should aim to confirm whether differences in outcomes between the two groups are present and determine the impact of the freezing process on sperm.

## Data Availability

The raw data supporting the conclusions of this article will be made available by the authors, without undue reservation.
